# Homology modeling and docking studies on oxidosqualene cyclases associated with primary and secondary metabolism of *Centella asiatica*

**DOI:** 10.1186/2193-1801-2-189

**Published:** 2013-04-27

**Authors:** Vadlapudi Kumar, Chethan S Kumar, Gajula Hari, Nayana K Venugopal, Poornima D Vijendra, Giridhara Basappa B

**Affiliations:** Department of Biochemistry, Davangere University, Shivagangothri, Davangere, Karnataka 577002 India

**Keywords:** Oxidosqualene cyclases, Cycloartenol synthase, β-amyrin synthase, Protein modeling, Docking studies

## Abstract

*Centella asiatica* is a well-known medicinal plant, produces large amount of triterpenoid saponins, collectively known as centelloids, with a wide-spectrum of pharmacological applications. Various strategies have been developed for the production of plant secondary metabolites in cell and tissue cultures; one of these is modular metabolic engineering, in which one of the competitive metabolic pathways is selectively suppressed to channelize precursor molecules for the production of desired molecules by another route. In plants the precursor 2,3-oxidosqualene is shared in between two competitive pathways involved with two isoforms of oxidosqualene cyclases. One is primary metabolic route for the synthesis of phytosterol like cycloartenol by cycloartenol synthase; another is secondary metabolic route for the synthesis of triterpenoid like β-amyrin by β-amyrin synthase. The present work is envisaged to evaluate specific negative modulators for cycloartenol synthase, to channelize the precursor molecule for the production of triterpenoids. As there are no experimentally determined structures for these enzymes reported in the literature, we have modeled the protein structures and were docked with a panel of ligands. Of the various modulators tested, ketoconazole has been evaluated as the negative modulator of primary metabolism that inhibits cycloartenol synthase specifically, while showing no interaction with β-amyrin synthase. Amino acid substitution studies confirmed that, ketoconazole is specific modulator for cycloartenol synthase, LYS728 is the key amino acid for the interaction. Our present study is a novel approach for identifying a suitable specific positive modulator for the over production of desired triterpenoid secondary metabolites in the cell cultures of plants.

## Introduction

Plant natural products and their derivatives play an important role in modern health care as frontline treatments for many diseases and as inspiration for chemical synthesized therapeutics (Pickens et al. 2011). *Centella asiatica* (L.) Urban, is a well-known medicinal plant, belongs to the family Apiaceae, has tremendous medicinal value and used as an important folk medicinal herb by natives of Asia, southern and middle Africa, southeastern United States and Australia, with a long history of therapeutic uses since ancient times. Preparations of *C. asiatica* are used in traditional and alternative medicine due to the wide spectrum of pharmacological activities. In common with most traditional phyto-therapeutic agents, *C. asiatica* is claimed to possess a wide range of pharmacological effects, being used for strengthening the weakened veins (Allegra 1981), wound healing (Sugana et al. 1996), mental disorders (Appa rao et al. 1973), atherosclerosis, fungicidal, antibacterial (Oyedeji & Afolayan 2005), sedative and anxiolytic (Kumar & Gupta 2002), antioxidant and anticancer purposes (Jayashree et al. 2003; Babu et al. 1995), antidepressant (Chen et al. 2003), antiepileptic (Hausen 1993), antinociceptive and anti-inflammatory (Somchit et al. 2004) and radio protective (Sharma & Sharma 2002). *C. asiatica* has also been reported to be a potent modulator of memory and hunger in both animals and humans, useful in the treatment of venous insufficiency, diarrhea, asthma, fever, improving cognition, tuberculosis and various skin lesions and aliments like leprosy, varicose ulcers, eczema, lupens, psoriasis, diarrhea and keloid (Gohil et al. 2010).

The immense medicinal properties of *c. asiatica* are attributed to the presence of secondary metabolites known as triterpenoid saponins. The plant contains large amount of triterpenoid saponins, collectively known as centelloids, includes asiaticoside, centelloside, madecassoside, brahmoside, brahminoside, thankuniside, sceffoleoside, centellose, asiatic-, brahmic-, centellic- and madecassic acids. The pharmacological and therapeutic applications of these triterpenes are mainly pentacyclic triterpenic acids and their respective glycosides, belonging to ursane- or oleanane-type, including asiatic acid, asiaticoside, madecassic acid, madecassoside, brahmoside, brahmic acid, brahminoside, thankuniside, isothankuniside, centelloside, madasiatic acid, centic acid, cenellic acid, betulinic acid, indocentic acid etc.

**Figure 1:**
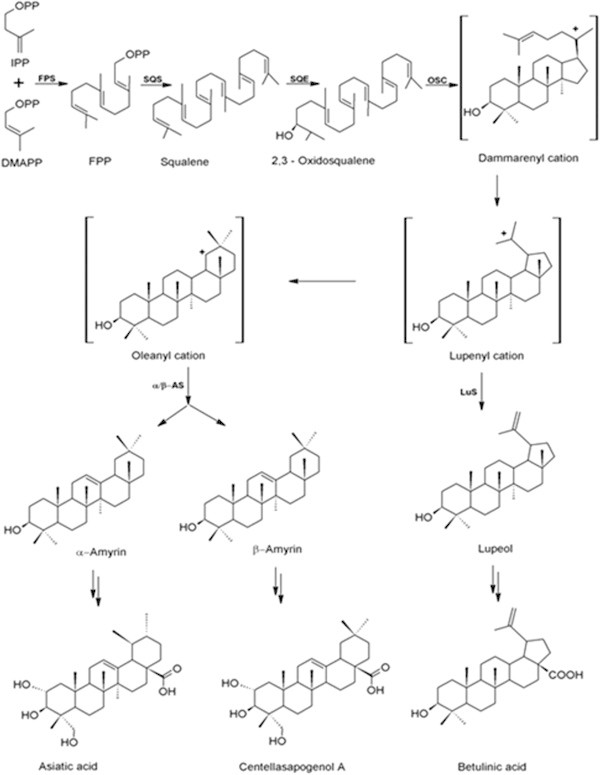
**A simplified scheme of triterpenoid biosynthesis in** ***C. asiatica.*** Farensyl diphosphate synthase (FPS) isomerizes isopentenyl diphosphate (IPP) and dimethylallyl diphosphate (DMAPP) to farensyl diphosphate (FPP), which squalene synthase (SQS) converts to squalene. Squalene epoxidase (SQE) oxidises squalene to 2,3-oxidosqualene. Oxidosqualene cyclase (OSC) enzymes cyclize 2,3-oxidosqualene through cationic intermendiates (e.g. dammarenyl cation) to one or more cyclic triterpene skeletons. Other enzymes involved include α/β-amyrin synthases (α/β-AS) which can also form the lupenyl cation but further ring expansion and rearrangements are required before the deprotonation to α/β-amyrin, the precursors of the sapogenins, to generate the products.

**Figure 2:**
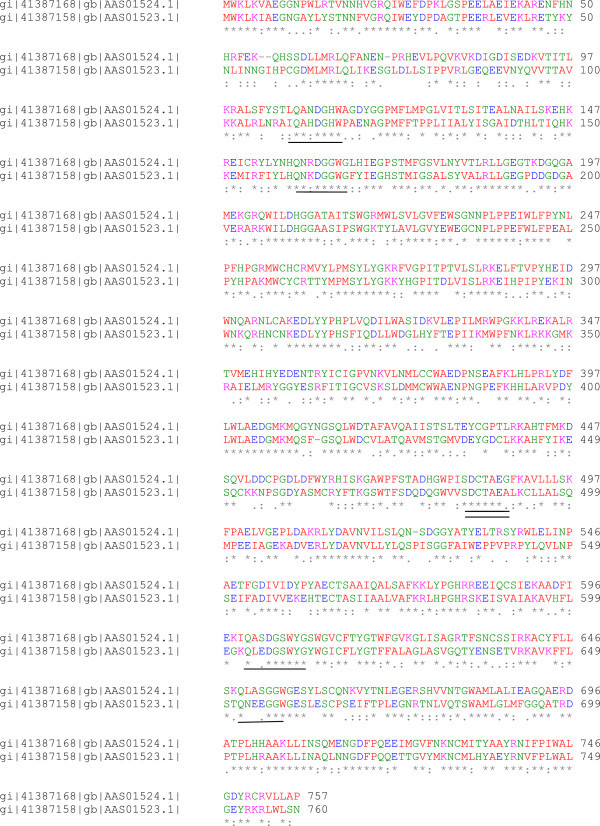
**Alignment of amino acid residues of** ***C. asiatica*** **cycloartenol synthase and β-amyrin synthase.** QW motifs are shown in single underline and DCTAE motifs in double underline.

In plants triterpenoids are synthesized via the isoprenoid pathway and derived from precursor 2,3-oxidosqualene, which is a common precursor molecule for both primary metabolites like plant sterols and secondary metabolites like triterpenoids (Phillips et al. 2006) (Figure 1). 2,3-oxidosqualene, the common precursor is transformed into either sterols or triterpenoids by oxidosqualene cyclases (OSCs) collectively known as triterpene synthases. Plants biosynthesize diverse triterpenoids and their genome encodes multiple OSC enzymes to form these skeletons. The level at which the structural diversity of triterpenes is generated depends on the cyclization of 2,3-oxidosqualene by different isoforms of OSCs such as cycloartenol synthase (CAS), lupeol synthase (LS) and α/β-amyrin synthase (AS) (Mangas et al. 2006). Cyclization of 2,3-oxidosqualene through a protosteryl cation intermediate generates lanosterol and cycloartenol, the structural precursors to all the sterols in plants, while cyclization through a dammerenyl, baccharenyl and lupeonyl cation intermediates generates lupeol and α/β-amyrin (Jenner et al. 2005) the precursors of the *Centella* pentacyclic triterpenoid saponins. The pertinent literature survey on sequence data of cycloartenol synthase (EC:5.4.99.8) (2,3-epoxysqualene--cycloartenol cyclase), that catalyzes the cyclization of (S)-2,3-epoxysqualene to cycloartenol, and β-amyrin synthase (EC 5.4.99.39) (2,3-epoxysqualene-- β-amyrin synthase), that catalyzes the cyclization of (S)-2,3-epoxysqualene to β-amyrin suggests that, the two enzymes isoforms and have several highly similar motiffs such as QW motif (Poralla et al. 1994) and DCTAE (Abe & Prestwich 1994) motifs, even though the reaction products are different for each of these cyclases (Figure 2). In spite of the sequence similarity, structural and functional diversity of plant oxidosqualene cyclases, not even a single oxidosqualene structure from the plant *per se* has been reported in the literature till-date, although functional diversity has been reported, as multifunctional OSCs do exist.

Plant secondary metabolites are incorporated into a wide range of commercial and industrial applications, and fortuitously, in many cases, rigorously controlled plant *in vitro* cultures can generate valuable natural products. There is great interest in developing alternatives to the intact plant for the production of secondary metabolites. The regular increasing demand in world marketplace for natural and renewable products has focused attention on *in vitro* plant materials as potential factories for phytochemical products, and has paved the way for new research exploring secondary product expression *in vitro*. In the recent years new approaches have been developed: the culturing of differentiated cells (e.g. shoots, roots), immobilized cell cultures, hairy root cultures induction by elicitors, tissue engineering and metabolic engineering (Anand 2010; Sevon et al. 1992; Sahai & Knuth 1985; Zhao et al. 2005; Zupan et al. 2000).

One of the approaches by metabolic engineering for the over production of desired metabolite is by blocking the competitive pathways (Verpoorte et al. 1994). Thus, by blocking flow of 2,3-oxidosqualene towards primary metabolism, it is possible to channelize the substrate to secondary metabolism by using suitable modulators that can inhibit primary metabolites-sterol biosynthesis. The aim of the present study is to evaluate and suggest suitable modulators that function like inhibitors for sterol biosynthesis, while without affecting the biosynthesis of triterpenoid secondary metabolites of *C. asiatica*. To achieve this, in the present study we made an attempt to build the protein structures of cycloartenol synthase (CAS) an enzyme associated with plant sterol (primary metabolite) biosynthesis and β-amyrin synthase (β-AS), an enzyme associated with plant triterpenoid saponin (secondary metabolite) biosynthesis, by homology modeling studies, and also to evaluate the specific interactions of these two enzymes with a panel of modulators by docking studies.

## Computational methods

Nucleotide sequences (cDNA) of cycloartenol synthase and β-amyrin synthase of *Centella asiatica* were retrieved from the NCBI database (http://www.ncbi.nlm.nih.gov/). These sequences were retrieved into FASTA format and used for further analysis. The modeling of the three dimensional structure of the protein was performed by using SWISS-MODEL (Arnold et al. 2006) (http://swissmodel.expasy.org/), the built model was visualized in molecular visualization software. Structural validation of protein was done using RAMPAGE (Lovell et al. 2002) (http://mordred.bioc.cam.ac.uk/~rapper/rampage.php), phi-psi torsion angles for all the residues in structure were plotted in the Ramachandran Plot at RAMPAGE.

Information regarding modulators of cycloartenol synthase (EC 5.4.99.8) and β-amyrin synthase (EC 5.4.99.40) were retrieved from BRENDA (http://www.brenda-enzymes.org) and also through data mining. These modulators were considered as ligands for the docking studies. Structures of modulators (ligands) were retrieved from PubChem (http://pubchem.ncbi.nlm.nih.gov/) and structures which are not available in the PubChem were drawn in ACD/ChemSketch. All the sdf and mol files obtained from the PubChem and ACD/ChemSketch were converted into pdb files using the Open Babel software. Prediction of ligand binding sites in the modeled protein structure was performed using Q-SiteFinder server (Laurie & Jackson 2005) (http://www.modelling.leeds.ac.uk/qsitefinder/), which were used in docking studies performed in Argus Lab. The modeled and docked structures were visualized in PyMol software.

In order to confirm the significance of LYS 728 in cycloartenol synthase and VAL 728 in β-amyrin synthase reciprocal studies were carried out by amino acid substitutions in the sequences of both cycloartenol synthase and β-amyrin synthase at position 728 residue, wherein cycloartenol synthase LYS 728 was substituted with VAL 728 and in β-amyrin synthase VAL 728 was substituted with LYS 728. Protein structures were modeled and ligand binding studies were carried out in SwissDock (http://www.swissdock.ch/), a free protein ligand docking web service powered by EADock DSS by the Molecular Modeling group of the Swiss Institute of Bioinformatics. The modeled and docked structures were visualized in PyMol software. Similarly modeling of the three dimensional structures both the proteins with substituted amino acid residues at position 728 were carried out using SWISS-MODEL (Arnold et al. 2006) (http://swissmodel.expasy.org/), the built model was visualized in molecular visualization software. Structural validation of protein was done using RAMPAGE (Lovell et al. 2002) (http://mordred.bioc.cam.ac.uk/~rapper/rampage.php), phi-psi torsion angles for all the residues in structure were plotted in the Ramachandran Plot at RAMPAGE.

## Results and discussion

**Figure 3:**
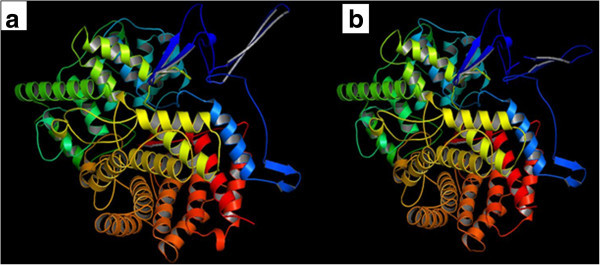
**Modelled structures of two enzymes of** ***C. asiatica*** **.** **a**) Cycloartenol synthase and **b**) β-amyrin synthase.

**Figure 4:**
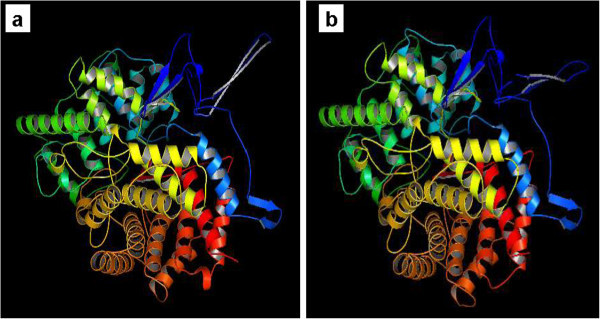
**Modelled structures of reciprocally amino acid substituted enzymes of** ***C. asiatica.*** **a**) Cycloartenol synthase and **b**) β-amyrin synthase.

**Figure 5:**
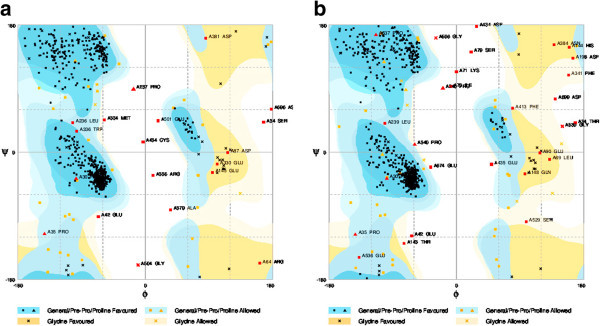
**Ramachandran Maps of CAS and β-AS generated by Rampage.** Dark blue and dark orange are favored regions. Light blue and light orange are allowed regions. **a**) Cycloartenol synthase and **b**) β-amyrin synthase.

**Figure 6:**
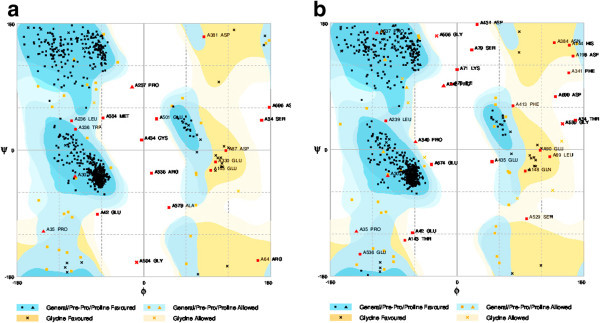
**Ramachandran Maps of CAS and β-AS generated by Rampage for reciprocally amino acid substituted enzymes of** ***C. asiatica*** **.** Dark blue and dark orange are favored regions. Light blue and light orange are allowed regions. **a**) Cycloartenol synthase and **b**) β-amyrin synthase.

The retrieved sequences of cycloartenol synthase and β-amyrin synthase of *Centella asiatica* for the present study are listed in Table 1. Results of protein modeling showed the QMEAN4 score of 0.42 and 0.45 (estimated model reliability between 0–1) taking human OSC (lanosterol synthase, PDB ID: 1W6J chain A) as a template structure and the similarities of were scored as 42.27% and 36.31% (Table 2). The obtained structures were visualized in PyMol (Figure 3). The stereo chemical quality of the predicted models and accuracy of the protein model was evaluated by Ramachandran Map calculations computed with the RAMPAGE and the results showed that 92.9% residues of cycloartenol synthase and 90.09% residues of β-amyrin synthase are in favoured region (Table 3, Figure 4). Similar results were obtained when validations were carried out with amino acid substitution at 728 position in both the enzymes reciprocally, with 99.9% of accuracy to that of amino acid non-substituted protein structures (Tables 4, 5 and Figures 5, 6).

**Table 1:** **Retrieved sequences from the databases**

Source	OSC	Accession no		Sequence length	
		Nucleotide	Protein	Nucleotide	Protein
*Centella asiatica*	Cycloartenol synthase	AY520819	AAS01524	2547 bp	757 aa
*Centella asiatica*	β-amyrin synthase	AY520818	AASO1523	2562 bp	760 aa

**Table 2:** **Results of protein modeling using SWISS-MODEL**

Template PDB ID	Target	Sequence identity	QMEAN4 Score
1W6JA	Cycloartenol synthase	42.27%	0.42
1W6JA	β-amyrin synthase	36.31%	0.45

**Table 3:** **Results of structure validation using RAMPAGE**

Assessment	Cycloartenol synthase	β-amyrin synthase
Favored region	92.90%	90.09%
Allowed region	4.50%	5.30%
Outlier region	2.50%	3.70%

**Table 4:** **Results of reciprocal studies of protein modeling using SWISS-MODEL**

Template PDB ID	Target	Sequence Identity	QMEAN4 Score
1W6JA	Cycloartenol synthase with VAL 728 for LYS 728	42.14%	0.424
1W6JA	β-amyrin synthase with LYS 728 for VAL 728	36.18%	0.405

**Table 5:** **Results of reciprocal studies of structure validation using RAMPAGE**

Assessment	Cycloartenol synthase with VAL 728 for LYS 728	β-amyrin synthase with LYS 728 for VAL 728
Favored region	92.90%	90.09%
Allowed region	4.50%	5.30%
Outlier region	2.50%	3.70%
Structure Accuracy with non-substituted structures	99.9%	99.9%

**Figure 7:**
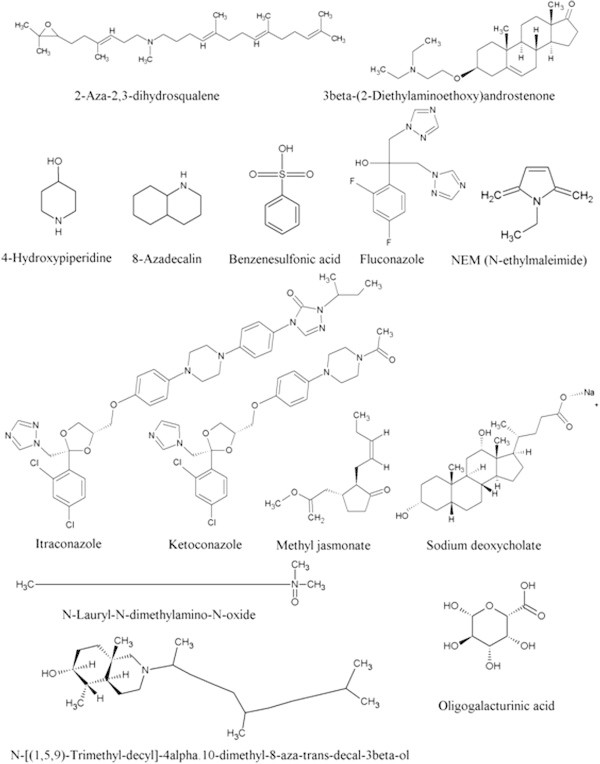
**Structures of the modulators considered for the study.**

Ligands which are considered for the present study as modulators (inhibitors/activators) for both cycloartenol synthase and β-amyrin synthase are listed in the Table 6 and structures are shown in Figure 7. The ligands bind at a specific site on enzymes; the binding site residues and their numbers are listed in the Table 7. Docking studies with ligands revealed that, the energy values of ligands, 2-aza-2,3-dihydrosqualene, 4-hydroxypiperidine, 8-azadecalin, benzenesulfonic acid, fluconazole, NEM (N-ethylmaleimide), N-[(1,5,9)-trimethyl-decayl]-4α,10-dimethyl-8-aza-trans-decal-3β-ol, N-lauryl-N-dimethylamino-N-oxide, methyl jasmonate, pectin/oligogalacturonic acid are non-specific and interact and inhibit both the enzymes (OSC isoforms) similarly. The modulators like 3β-(2-diethylaminoethoxy) aldosterone and sodium deoxycholate did not show any interactions with cycloartenol synthase, whereas they showed affinity to bind β-amyrin synthase, hence these ligands could be considered as β-amyrin synthase specific modulators. While, itraconazole failed to interact with either of the enzymes.

**Table 6:** **Modulators for cycloartenol synthase and β-amyrin synthase**

Sl. No.	Modulators used in the present study
1	2-aza-2,3-dihydrosqualene^a^
2	3-β-(2-Diethylaminoethoxy) androsteroneandrosterone^b^
3	4-hydroxypiperidine^c^
4	8-azadecalin^c^
5	Benzenesulfonic acid^d^
6	Fluconazole^e^
7	Itraconazole^e^
8	Ketoconazole^e^
9	Methyl jasmonate^f^
10	NEM (N-ethylmaleimide)^g^
11	Sodium deoxycholate^h^
12	N-[(1,5,9)-Trimethyl-decayl]-4α, 10-dimethyl-8-aza-trans-decal-3β-ol^i^
13	N-lauryl-N-dimethylamino-N-oxide^j^
14	Pectin/Oligogalacturonic acid^k^

^a^ Ref : (Delprino et al. 1983).

^b^ Ref : (Fenner & Raphiou 1995).

^c^ Ref : (Taton et al. 1992).

^d^ Ref : (Abe et al. 1993).

^e^ Ref : (Goldman et al. 1996).

^f^ Ref : (Kim et al. 2005).

^g^ Ref : (Abe et al. 1992).

^h^ Ref : (Beastall et al. 1971).

^i^ Ref : (Taton et al. 1986).

^j^ Ref : (Schmitt et al. 1987).

^k^ Ref : (Flores-Sanchez et al. 2002; Hu et al. 2003).

**Table 7:** **Predicted ligand binding site of CAS and β-AS using Q-SiteFinder**

Cycloartenol synthase		β-amyrin synthase	
Residues	Residue number	Residues	Residue number
TYR	118	PRO	124
PRO	121	PHE	127
LEU	124	TRP	258
TRP	255	CYS	259
CYS	256	TYR	260
HIS	257	ILE	368
CYS	258	GLY	369
ILE	365	CYS	370
GLY	366	VAL	771
PRO	367	GLN	411
VAL	368	SER	412
GLY	409	TRP	418
TYR	410	PHE	474
TRP	479	VAL	483
ILE	481	ASP	485
THR	531	CYS	486
TYR	532	ILE	534
GLU	533	TRP	535
PHE	550	CYS	565
ILE	553	TRP	613
ASP	556	TYR	619
TYR	559	*VAL	*728
SER	609	TYR	729
TRP	610	LEU	735
VAL	725	TYR	737
PHE	726	TYR	740
ASN	727		
*LYS	*728		
CYS	730		
ILE	732		

*Amino acid residue changes between two enzymes at ketoconazole binding site of cycloartenol synthase (LYS 728).

**Figure 8:**
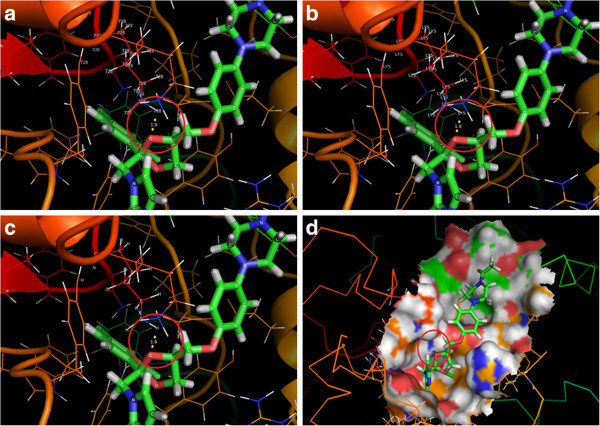
**Interaction between CAS and ketoconazole in cartoon and solid representation.** (**a**) Interaction between amino acid residue 728 and hydrogen atom of ketoconazole. (**b**) Interaction between amino residue LYS 728 of CAS and hydrogen atom of ketoconazole. (**c**) Interaction between hydrogen atom of CAS and oxygen atom of ketoconazole. (**d**) Binding surface of CAS.

Of all the modulators tested, ketoconazole has been evaluated as a negative modulator for primary metabolism of *C. asiatica*, as the molecule specifically inhibits cycloartenol synthase (E = −8.82927 kcal/mol), but failed to interact with β-amyrin synthase. Hence, this ligand ketoconazole could be considered as a specific inhibitor of cycloartenol synthase, and therefore could suppress or control primary metabolism i.e., sterol biosynthesis, and could channel the substrate 2,3-oxidosqualene for secondary metabolite (triterpenoid) biosynthesis, thus could enhance the secondary metabolism (Table 8). Ketoconazole interacts specifically with LYS 728 of cycloartenol synthase (Figure 8a). The interactions between the hydrogen atom in the LYS 728 and the oxygen atom in the ketoconazole were confirmed to be hydrophilic interactions (Figure 8a-d).

**Table 8:** **Docking results of CAS and BAS using Argus Lab**

Ligands	Best ligand pose (E = kcal/mol)	
	Cycloartenol synthase	β-amyrin synthase
2-Aza-2,3-dihydrosqualene	−19.1334	−19.4249
3-β-(2-Diethylaminoethoxy) androsteroneandrosterone	---*	−13.1427
4-hydroxypiperidine	- 6.96723	- 7.01001
8-Azadecalin	- 9.3033	- 10.027
Benzenesulfonic acid	- 9.19979	- 10.1391
Fluconazole	- 9.44939	- 10.1095
Itraconazole	---*	---*
Ketoconazole	- 8.82927	---*
NEM (N-ethylmaleimide)	- 6.36966	- 6.89406
Sodium deoxycholate	---*	- 14.3559
N-[(1,5,9)-Trimethyl-decayl]-4alpha, 10-dimethyl-8-aza-trans-decal-3beta-ol	- 15.4413	- 15.4102
N-Lauryl-N-dimethylamino-N-oxide	- 11.4094	- 12.2249
Pectin/Oligogalacturonic acid	- 7.60546	- 7.48043
Methyl jasmonate	- 11.2485	- 11.2796

---* No acceptable ligand poses were found.

**Figure 9:**
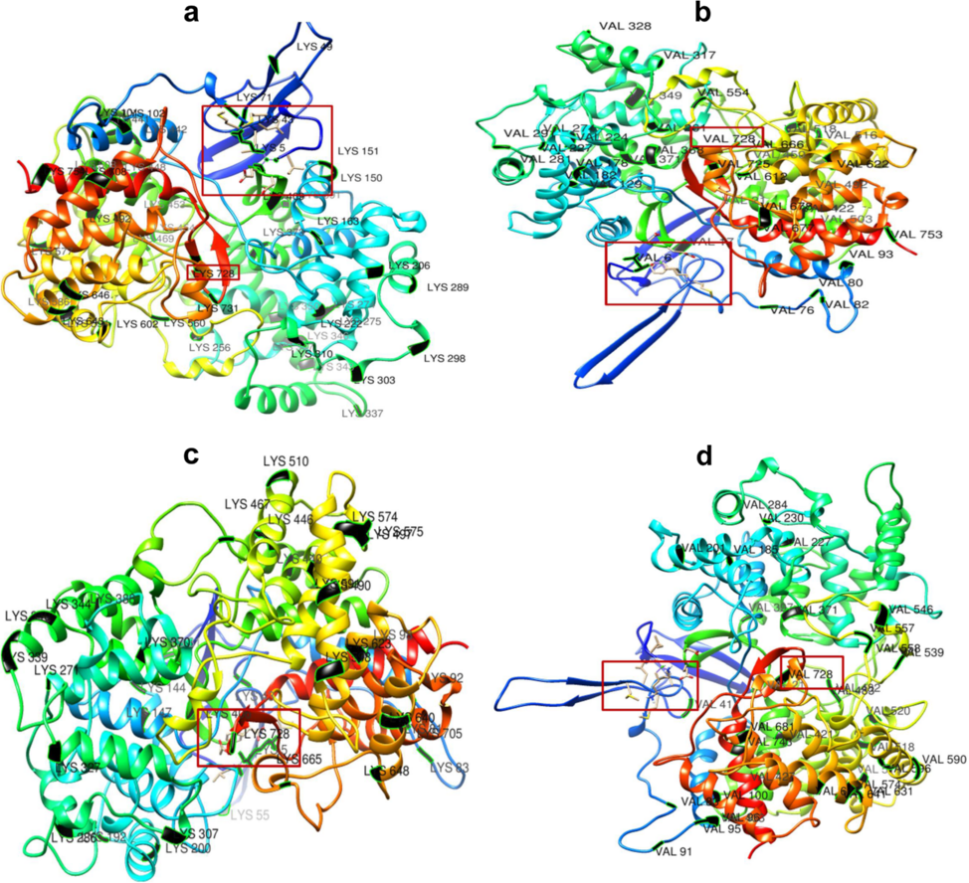
**Interaction between reciprocally amino acid substituted enzymes of** ***C. asiatica*** **.** (**a**) Non-substituted CAS with LYS 728 and ketoconazole. (**b**) Substituted CAS with VAL 728 for LYS 728 and ketoconazole. (**c**) Non-substituted BAS with VAL 728 and ketoconazole. (**d**) Substituted BAS with LYS 728 for VAL 728 and ketoconazole. *Ligand and amino acids at 728 position in modeled protein structures are shown in rectangle/square.

Reciprocal studies were carried out with amino acid substitutions at 728 position in the amino acid sequences of cycloartenol synthase LYS 728 was substituted with VAL 728 and β-amyrin synthase VAL 728 was substituted with LYS 728, protein models were docked with ketoconazole, using SwissDock software. Results of docking studies carried out with SwissDock using ketoconazole with normal and amino acid substituents of both cycloartenol synthase and β-amyrin synthase revealed that, ketoconazole could specifically interact with LYS 728 of normal cycloartenol synthase only and failed to interact with cycloartenol synthase containing VAL 728 for LYS 728, normal β-amyrin synthase and even with β-amyrin synthase containing LYS 728 for VAL 728 (Figure 9a-d). This confirms the specificity of ketoconazole interaction with cycloartenol synthase.

Ketoconazole is an azole fungicide, inhibits both fungal and mammalian cytochrome P450 oxidases (CYPs) that are associated with sterol metabolism. At concentrations >100 nM, ketoconazole inhibits both fungal and mammalian CYP51s, that play an important role in ergosterol and cholesterol biosynthesis respectively, and also affect the activity of enzymes involved in catabolism of cholesterol. More specifically, ketoconazole inhibits 17-hydroxylase-17,20-lyase (CYP17), the cholesterol side chain cleavage enzyme (CYP11A1), and the 11-β-hydroxylase (CYP11B1) (Vanden 1992). The 50% inhibitory concentration (IC_50_) of ketoconazole for lanosterol synthase was elucidated to be 11.7 nM (Sakaeda et al. 2005). Of the two oxidosqualene cyclases investigated in the present study, cycloartenol synthase is considered to be a plant equivalent for cholesterol synthesis in animals and ergosterol synthesis in fungi. The cyclization is executed with a remarkable degree of specificity and stereochemical control to produce protosterol intermediates. The two ‘protosterols’ that are subsequently modified to functional products such as cholesterol or phytosterols. The products are either lanosterol (in animals and fungi) or cycloartenol (in plants). The two enzymes mediate the cyclization process identically until the final deprotonation step. A deprotonation from C9 forms the 8,9-double bond of lanosterol whereas a deprotonation from C19 allows the cycloartenol cyclopropyl ring to close. Thus far, lanosterol synthase has been found only among the opisthokonts (animals + fungi + choanozoa), trypanosomatids (*Trypanosoma, Leishmania*) and dinoflagellates (Giner et al. 1991; Roger et al. 2006). All other eukaryotes that have been examined in this regard (at least members of the higher plants, green and red algae, amoebozoa, diatoms, euglenids and heterolobosea) make cycloartenol as their protosterol (Roger et al. 2006). Results of ligand binding site analysis using Q-SiteFinder revealed that, ketoconazole interaction with cycloartenol synthase is hydrophilic interaction with Lys 728 (hydrophilic amino acid) at the active site, whereas it is substituted with Val 728 (hydrophobic amino acid) at the same position in β-amyrin synthase. This hydrophilic-to- hydrophobic substitution of single amino acid residue at the enzyme active site probably distinguishes the two OSC isoforms to show distinctive interaction specificity with the ligand ketoconazole. This has also been proved through reciprocal amino acid substitution studies in both the enzymes.

## Conclusion

The *in vitro* plant enzyme modulator studies are long and expensive one. It starts from target identification, after that, validates the targets and identifies modulators. Due to the limitation of throughput, accuracy and cost, experimental techniques cannot be applied widely; therefore, our study has shifted to *in silico* approaches such as homology modeling and protein-ligand interactions. *In silico* approach has been of great importance as a versatile tool to develop fast and accurate target identification and prediction method for the discovery. The present work is an attempt to identify a specific modulator that would control the primary metabolism and over produce the secondary metabolites by channeling the precursor/substrate in *Centella asiatica* cell cultures because of its immense medicinal importance. The docking studies particularly with ketoconazole has explored the fact that, the two oxidosqualene isoforms differ from each other by virtue of a single amino acid substitution at 728 position from lysine to valine. The results of the present study, suggest that, because of its hydrophilic interaction, ketoconazole can possibly channelize the precursor molecule 2,3-oxidosqualene towards secondary metabolism by functioning like a negative modulator of sterol biosynthesis, and at the same time as a positive modulator for the over production of triterpenoid secondary metabolites in not only cell cultures *Centella asiatica* of other plants too. To prove this, in our lab we have initiated studies using the cell suspension cultures of *Gymnema sylvestre*, for the overproduction of gymnemic acid, a group of triterpenoid saponins.
